# To Our Patrons

**Published:** 1856-10

**Authors:** 


### TO OUR PATRONS.

We must take the liberty of again calling the attention of our subscribers to arrearages due for subscription. We do not ask confidence in our pretentions till we give evidence which we think should warrant it. We know that Journals have *failed*, and cash subscribers have been *squeezed;* therefore, although cash terms were advertised, subscribers were not held strictly to cash during the publication of the first volume. Now, as this is the 2d No. of the 2d Vol., we hope that our friends, who are in arrears, will remember us *pecuniarily*, and remit for vols. 1 and 2, if convenient. We know the feelings of subscribers. We have been, and are now subscribers. The amount is small, too small to engage much thought in those owing these individual and separate amounts. Yet, think, friends! the aggregate of these separate small amounts is to pay the publication of a Journal which costs, in cash, every month, the sum of about ninety dollars. With our present list of subscribers, this monthly debt could be easily discharged, without inconvenience to our patrons. Therefore, brethren, send in these small amounts, for which we hope you have value received, and relieve the Faculty, under whose control the Journal is published, from a burthen they have patiently shouldered for twelve months. We are willing to contribute the labor editorial, which is no small matter, without the hope of pecuniary remuneration, and ask only that due subscriptions, sufficient to pay the publication, be sent in.

